# Menopause matters: A community-based cross-sectional online survey among midlife women in Malaysia

**DOI:** 10.51866/oa.1006

**Published:** 2026-06-04

**Authors:** Rara Merinda Puspitasari, Azizatul Munirah Zaini, Zaswiza Mohamad Noor, Syarifah Syamimi Putri Adiba Syed Putera

**Affiliations:** 1 Faculty of Pharmacy and Health Sciences, Royal College of Medicine Perak, Universiti Kuala Lumpur, Ipoh, Perak, Malaysia.

**Keywords:** Menopause, Health knowledge, Attitudes, Practice, Middle aged, Malaysia

## Abstract

**Introduction:**

Menopause is a substantial life transition for women, signifying the end of reproductive capability, with implications for symptoms, long-term health and well-being. Women’s experiences during menopause can differ widely, affecting their attitudes towards this transition. This study aimed to investigate knowledge and attitudes towards menopause among midlife women in Malaysia.

**Methods:**

A community-based cross-sectional online survey using non-probability sampling was conducted among Malaysian women aged 40–60 years residing in an urban setting. A validated online questionnaire was used to assess the study parameters. Knowledge and attitudes were quantified, and associations with sociodemographic factors were examined using the Kruskal-Wallis test. Descriptive statistics and Spearman’s correlation analysis were performed using the IBM Statistical Package for the Social Sciences Statistics version 26.0.

**Results:**

Of the 300 respondents, the majority were Malay (94.7%), married (85.3%) and aged 55–60 years (27.3%). Over half (52.0%; mean score=12.98±6.77 [22 max score]) demonstrated poor knowledge, while 45.3% held negative attitudes towards menopause. The average attitude score was 33.48±6.58 [56 max score], indicating a generally negative orientation towards menopause. A weak but significant positive correlation was observed between knowledge and attitudes (r=0.249, P<0.01).

**Conclusion:**

Targeted educational initiatives may be beneficial in enhancing menopause-related knowledge and fostering more positive attitudes among midlife Malaysian women. Future research should rigorously assess changes in knowledge, attitudes and related clinical behaviours after such interventions to inform policy and practice.

## Introduction

Midlife, typically spanning the ages of 40–65 years, represents a crucial transition period in a woman’s life, which encompasses numerous physiological, psychological and social changes. Among these changes, menopause stands out as a major milestone, signifying the permanent cessation of menstruation and the end of natural reproductive capability.^1^ Globally, menopause most commonly occurs within the ages of 45–55 years, although the precise age varies with ethnicity, genetics and environmental factors.^2,3^

Malaysia, a multicultural and multi-ethnic Southeast Asian country, has a diverse population, with midlife women constituting a substantial portion of the population.^4^ Although the prevalence of menopause in Malaysia is not extensively recorded, studies estimate the average age at menopause to be around 49.9–50.7 years.^5-8^ However, previous Malaysian studies have largely focused on menopausal symptoms or clinical populations, with limited research examining women’s knowledge and attitudes towards menopause in community settings.

The menopausal transition may impact women’s quality of life, leading to various physical, psychological and social changes. It is marked by declining ovarian function and reduced oestrogen production. The hormonal shift is associated with a wide spectrum of symptoms, including vasomotor symptoms (e.g. hot flashes and night sweats), genitourinary changes (e.g. vaginal dryness and urinary frequency), physiological symptoms (e.g. mood swings, depression and anxiety), sleep disturbances, stress, exhaustion, dry skin, musculoskeletal pain and cognitive complaints.^8-13^ Beyond physical symptoms, menopause also increases the risk of osteoporosis, cardiovascular disease and metabolic disorders, posing important challenges to women’s long-term health and well-being.^14^

Women’s knowledge about these changes and their attitudes towards menopause influence their health-seeking behaviours, symptom management and quality of life.^10^ Adequate knowledge enables women to recognise menopausal symptoms and supports appropriate self-management and help-seeking when needed.^15^ In addition to knowledge, attitudes towards menopause reflect women’s beliefs, perceptions and emotional responses to the menopausal transition.^16^ Women’s attitudes may be both positive and negative, depending on how menopause is perceived.^10,17-19^ While some view it as a normal phase that must be endured, others perceive it as a burden, leading to a negative perception or attitude. Women who exhibit a positive attitude towards menopause express no regret over the cessation of menses. However, some of them view menopause negatively, seeing it as a time when their beauty, youth and femininity diminish with age.^10,19-21^

Accumulating evidence highlights a positive correlation of menopause-related knowledge and attitudes with effective symptom management. Studies in various populations have demonstrated that increased awareness is associated with more adaptive attitudes, greater acceptance and proactive symptom management.^10,22,24^

Midlife women’s knowledge and attitudes towards menopause can significantly influence their experiences during this life transition. Investigating awareness of menopausal processes and the perceived significance of menopause can provide insights into the need for education and awareness campaigns. Although a few studies in Malaysia have assessed women’s knowledge and awareness of menopause, the majority have focused on the prevalence of menopausal symptoms and sociocultural aspects.^5,9,25-27^ Additionally, cultural taboos and lack of open discussion may further limit women’s ability to access reliable information and support during this transition.^7,9,27^

Understanding the knowledge and attitudes of midlife Malaysian women towards menopause is thus essential for developing effective educational interventions, informing healthcare policy and ultimately improving women’s health outcomes. This study aimed to address this gap by investigating menopause-related knowledge and attitudes among Malaysian women aged 40-60 years and associated sociodemographic factors. The findings will inform the development of targeted strategies to empower women, reduce stigma and support healthy ageing in the Malaysian context.

## Methods

### Study design and participants

A cross-sectional online survey was conducted among Malaysian women aged 40-60 years residing in Ipoh, the capital city of Perak, located in the northern region of Peninsular Malaysia. The study targeted women from the general community across the menopausal spectrum, including premenopausal, perimenopausal and postmenopausal women. Women were excluded when they reported menopause induced by surgical or medical causes as well as premature menopause. The minimum sample size was calculated using a confidence level of 95%, a population proportion of 20.5% (representing the proportion of women aged 40-64 years in Malaysia) and a margin of error of 5%. The resulting required sample size was 242 participants.^28^

### Data collection

A non-probability sampling approach combining purposive and snowball sampling was used. Data were collected using a structured questionnaire administered via Google Forms, which included a cover letter and an electronic informed consent form. The survey link was distributed through social media platforms, including Facebook, Twitter and WhatsApp. The invitation to participate was shared via the researchers’ personal social media accounts for public posting as well as through direct messaging to contacts and community-based online groups, with participants encouraged to forward the survey link to other eligible women. Data collection was conducted over 4 months.

Participation was voluntary, and no incentives were offered. Respondents’ confidentiality and anonymity were assured. Collected data were used solely for scientific purposes.

### Study questionnaire

The questionnaire was developed by adapting and selecting items from previously published studies that assessed similar parameters. A content validation process was conducted, in which two panels of experts reviewed and evaluated the questionnaire for relevance, clarity and appropriateness. The content validity index was calculated, with all items achieving values greater than 0.80, meeting the recommended threshold.

A pilot study involving 30 participants was conducted to assess the reliability of the questionnaire. Pilot participants were recruited from the researchers’ networks using the same online recruitment approach as the main study. The internal consistency analysis demonstrated good reliability, with a Cronbach’s alpha value of 0.916.

The questionnaire comprised three parts:

1. *Sociodemographics:* Age, ethnicity, educational level, income and related characteristics.
2. *Knowledge of menopause:* The knowledge section evaluated understanding of the definition of menopause, age of onset, hormonal changes, fertility, hormone replacement therapy (HRT), common symptoms and menopause-related health risks. These items were adapted from previously published questions about knowledge, attitude and practice (KAP) towards menopause.^10,11,29,30^ The section comprised 22 items. Each correct answer was counted as 1 point and each incorrect answer as 0 points. The total score ranged from 0 to 22. The knowledge scores were categorised using Bloom’s cut-off point.^31^ Based on the percentage of correct responses, the knowledge levels were classified as good (≥18), moderate (13–17) and poor (≤12).
3. *Attitudes towards menopause:* The attitude section assessed women’s perceptions and emotional responses towards menopause using a 5-point Likert scale. Items were adapted from validated attitude-based studies examining psychosocial views of menopause.^10,30,32,33^ Modifications were made to ensure cultural relevance. The section comprised 14 Likert-scale items (scored 0–4). Response options included *strongly disagree* (0), *disagree* (1), *neutral* (2), *agree* (3) and *strongly agree* (4). The minimum total score was 0, while the maximum score was 56. Scores of ≥34 (61% of the total score) indicated positive attitudes. A ≥60% cut-off was used to define positive attitudes, consistent with conventional KAP scoring methods and representing scores above the neutral midpoint in the absence of a validated standard threshold.

### Definition of terms

For the purpose of this study, the following operational definitions were applied. Educational background was categorised into three groups: (i) health science/medical, referring to participants with formal education or training in health-related fields such as medicine, nursing, pharmacy, allied health sciences or biomedical sciences; (ii) non-health science, referring to participants with education in fields not related to health or medical sciences; and (iii) not applicable, referring to participants who did not report any formal tertiary education, including those with no formal education and those whose highest educational attainment was primary or secondary education.

Residence referred to participants’ place of living at the time of the study and was categorised as urban or non-urban (suburban/rural) based on self-report.

Menstrual status was classified based on participants’ self-reported menstrual history and categorised as premenopausal (regular menstrual cycles), perimenopausal (irregular menstrual cycles or menopausal symptoms with menstruation within the past 12 months) and postmenopausal (absence of menstruation for at least 12 consecutive months).

### Data analysis

Data were analysed using the Statistical Package for the Social Sciences (SPSS) version 26.0 (SPSS Inc, Chicago, IL). Descriptive statistics were used to summarise sociodemographic characteristics and study variables, which were presented as frequencies and percentages. As the knowledge and attitude scores were not normally distributed, non-parametric tests were applied. Associations between menopause-related knowledge and attitude scores and sociodemographic factors were examined using the Kruskal–Wallis test. Post-hoc pairwise comparisons were not performed following the Kruskal–Wallis test, as the objective of the analysis was to examine overall associations rather than specific between-group differences. Correlations between knowledge and attitude scores were assessed using Spearman’s rank-order correlation. A P-value of <0.05 was considered statistically significant.

## Results

### Respondent characteristics

A total of 313 responses were collected from Google Forms, with 300 meeting the inclusion criteria and undergoing data analysis. Thirteen respondents were excluded due to a history of chemotherapy, radiotherapy or surgical procedure to remove the ovaries. The Kolmogorov–Smirnov test indicated that the data on all respondent characteristics were not normally distributed (P<0.05).

Table 1 summarises the respondent characteristics. Most respondents were Malay, making up 94.7% of the total. Approximately 69% had a higher educational level, with 73.3% having no educational background in health sciences or non-health sciences. Additionally, the majority belonged to the M40 income group (RM 4360–9619), and 45.3% resided in urban areas. Most respondents were married (85.3%). Nearly half (43.6%) were still in the premenopausal stages, while 30.6% were in the postmenopausal stages.

**Table 1:** Respondent characteristics.

Questionnaire item	n (N=300)	%
**Age, year**		
40-44	62	20.7
45-49	81	27.0
50-54	75	25.0
55-60	82	27.3
**Ethnicity**		
Malay	284	94.7
Chinese	9	3.0
Indian	7	2.3
**Marital status**		
Unmarried	17	5.7
Married	256	85.3
Widowed/divorced	27	9.0
**Highest educational level**		
No formal education	7	2.3
Primary education	8	2.7
Secondary education	78	26.0
Higher education	207	69.0
**Educational background**		
Related to health science/medical	23	7.7
Non-health science	57	19.0
Not applicable	220	73.3
**Occupational status**		
Working (full-time)	209	69.7
Working (part-time)	15	5.0
Housewife	50	16.7
Retired	26	8.6
Family income		
B40 (<RM 4360)	81	27.0
M40 (RM 4360-9619)	154	51.3
T20 (>RM 9619)	65	21.7
**Residence**		
Urban	136	45.3
Suburban	114	38.0
Rural	50	16.7
**Menstrual status**		
Premenopausal	131	43.7
Perimenopausal	57	19
Menopausal	20	6.7
Postmenopausal	92	30.6

**Table 2:** Knowledge of menopause.

No.	Questionnaire item	N=300 (100%)	
		Correct, n (%)	Incorrect, n (%)
	**General knowledge of menopause**		
1	Definition of menopause	91 (30.3)	209 (69.7)
2	Average age at menopause	126 (42.0)	174 (58.0)
3	Menopause may occur earlier in women with poor health.	200 (66.7)	100 (33.3)
4	Menopause may occur due to increasing sexual hormone levels.	95 (31.7)	205 (68.3)
5	Menopausal symptoms are preventable and curable.	135 (45.0)	165 (55.0)
6	After regular menstruation stops, pregnancy prevention is not necessary.	226 (75.3)	74 (24.7)
7	Hormone replacement therapy is prescribed to ease menopaus­al symptoms.	188 (62.7)	112 (37.3)
	**Symptoms of menopause**		
8	Hot flashes in the upper body (face, neck or chest)	186 (62.0)	114 (38.0)
9	Mood swings	256 (85.3)	44 (14.7)
10	Vaginal dryness	214 (71.3)	86 (28.7)
11	Night sweats	189 (63.0)	111 (37.0)
12	Thinning hair and dry skin	206 (68.7)	94 (31.3)
13	Sleep problems	190 (63.3)	110 (36.7)
	**Associated risks of menopause**		
14	Increased risk of cancer especially breast cancer	131 (43.7)	169 (56.3)
15	Increased risk of osteoporosis	207 (69.0)	93 (31.0)
16	Increased risk of diabetes	129 (43.0)	171 (57.0)
17	Difficulty concentrating or inability to concentrate	155 (51.7)	145 (48.3)
18	Decreased memory	162 (54.0)	138 (46.0)
19	Increased risk of genital (vaginal) infections	141 (47.0)	159 (53.0)
20	Increased risk of cardiovascular disease	140 (46.7)	160 (53.3)
21	Increased risk of stress and depression	184 (61.3)	116 (38.7)
22	Weight gain and increased body fat	178 (59.3)	122 (40.7)

### Knowledge of menopause

Table 2 displays the respondents’ knowledge of menopause. The respondents were asked about their general knowledge regarding menopause (e.g. description, average age of onset and general facts), symptoms of menopause and associated risks of menopause. Most respondents (52.0%) demonstrated poor knowledge of menopause, while 29.3% and 18.7% had good and moderate knowledge, respectively. The mean total knowledge score was 12.98±6.77 out of a total possible score of 22.

Only 30.3% of the respondents defined menopause correctly as 12 consecutive months without a menstruation. Approximately 42.0% correctly identified the average age at menopause as 51 years. Additionally, 66.7% were aware that menopause may occur earlier in women with poor health. A total of 68.3% of the respondents knew that menopause does not result from increased sexual hormone levels, but 55.0% were unaware that menopausal symptoms are preventable and curable. The majority were knowledgeable that after regular menstruation stops, pregnancy prevention is no longer required (75.3%) and that HRT is prescribed to alleviate menopausal symptoms (62.7%).

Knowledge regarding the symptoms of menopause was assessed using questions 8–13. Over 60% of the respondents were aware that hot flashes in the upper body (facial, neck or chest), mood swings, vaginal dryness, night sweats, thinning hair, dry skin and sleep problems are signs and symptoms of menopause.

Questions 14–22 addressed the health-related risks associated with menopause. A large number of the respondents were unaware that menopause increases the risk of cancer, particularly breast cancer (56.3%), diabetes (57.0%) and cardiovascular diseases (53.3%). However, approximately 69% recognised the increased risk of osteoporosis, while about half were aware of risks such as vaginal infections (53.0%), decreased memory (54.0%) and difficulty concentrating (51.7%).

### Attitudes towards menopause

Table 3 presents the analyses of the responses to the 14 questions used to assess attitudes towards menopause. These questions explored the respondents’ perspectives, feelings and preconceived notions about menopause, which were either positive, negative or neutral. Questions 1–6 encompassed negative attitude statements, while questions 7–14 captured positive attitude statements.

**Table 3:** Attitudes towards menopause.

Questionnaire item	Strongly disagree n (%)	Disagree n (%)	Neutral n (%)	Agree n (%)	Strongly agree n (%)
I feel anxious about getting menopause because it is the end of youth for women.	22 (7.3)	68 (22.7)	107 (35.7)	66 (22.0)	37 (12.3)
I feel afraid because menopause is the beginning of an unproductive life.	32 (10.7)	75 (25.0)	108 (36.0)	55 (18.3)	30 (10.0)
Menopause is an unpleasant experience in my life.	32 (10.7)	82 (27.3)	109 (36.3)	55 (18.3)	22 (7.3)
I feel bothered by menopause because it is the beginning of distress in a woman’s life.	32 (10.7)	78 (26.0)	106 (35.3)	57 (19.0)	27 (9.0)
I feel mentally and emotionally drained at the time of menopause.	22 (7.3)	69 (23.0)	108 (36.0)	71 (23.7)	30 (10.0)
After menopause, I do not consider myself a real woman.	55 (18.3)	73 (24.3)	112 (37.3)	33 (11.0)	27 (9.0)
Going through menopause does not change myself much in terms of daily life.	9 (3.0)	13 (4.3)	129 (43.0)	94 (31.3)	55 (18.3)
Menopause makes my life easier because I do not have to use sanitary pads anymore.	9 (3.0)	12 (4.0)	108 (36.0)	107 (35.7)	64 (21.3)
Menopause will ease my life because I will not experience menstrual pain and problems anymore.	9 (3.0)	12 (4.0)	122 (44.7)	95 (31.7)	62 (20.7)
I feel relieved because I no longer need to use birth control methods.	9 (3.0)	13 (4.3)	119 (39.7)	102 (34.0)	57 (19.0)
At the time of menopause, I will no longer experience distress due to irregular menses.	8 (2.7)	15 (5.0)	125 (41.7)	97 (32.3)	55 (18.3)
I feel relieved that I do not have to worry about the risk of getting pregnant after getting menopause.	10 (3.3)	12 (4.0)	114 (38.0)	101 (33.7)	63 (21.0)
My life will become more interesting after menopause.	6 (2.0)	15 (5.0)	156 (52.0)	80 (26.7)	43 (14.3)
I am not scared of menopause because it is a normal period in women’s life.	9 (3.0)	11 (3.7)	94 (31.3)	113 (37.7)	73 (24.3)

The total possible attitude score ranged from 0 to 56. A total score below 34 indicated a negative attitude, while a score above 34 suggested a positive attitude. More than half of the respondents (55%) have a negative attitude, whereas 45% (n=136) have positive attitude toward menopause. The mean total attitude score among the respondents was 33.48±6.58, indicating that, on average, they had more negative attitudes towards menopause.

Approximately 20.0%–34.3% of the respondents had negative attitudes towards menopause, citing anxiety due to its representation as the end of a woman’s youth and the commencement of an unproductive life. They perceived menopause as an unpleasant experience, anticipating distress and feeling mentally and emotionally depleted during this period.

A large number of the respondents (35.5%–37.3%) had neutral responses to the negative attitude statements. Similarly, about 31.3%–52.0% provided neutral responses to the positive attitude statements. However, the percentage of the respondents who agreed with the positive attitude statements was higher, ranging from 41.0% to 62.0%, than the percentage of those who agreed with the negative attitude statements.

### Association of the respondent characteristics with knowledge and attitudes

The association of the respondent characteristics with knowledge and attitudes is presented in Table 4. The demographic characteristics showed non-normal distribution, so the P-values were generated using the Kruskal–Wallis test and the means and standard deviations using a non-parametric test.

**Table 4:** Association of the respondent characteristics with knowledge and attitudes.

Description	N=300; n (%)	Knowledge		Attitude	
		Mean±SD	P-value	Mean±SD	P-value
Age, year					
40-44	62 (20.7)	10.710±0.984	**0.029**	31.419±0.585	**0.007**
45-49	81 (27.0)	13.407±0.822		32.765±0.655	
50-54	75 (25.0)	14.160±0.719		33.764±0.779	
55-60	82 (27.3)	13.207±0.607		35.500±0.854	
Marital status					
Unmarried	17 (5.7)	10.529±1.851	0.081	29.412±0.600	**0.011**
Married	256 (85.3)	13.332±0.417		33.828±0.419	
Widowed	17 (5.7)	9.941±1.510		33.470±1.585	
Divorced	10 (3.3)	13.400±2.363		31.500±1.857	
Highest educational level					
No formal education	7 (2.3)	14.857±2.995	**<0.001**	29.857±0.911	0.336
Primary education	8 (2.7)	7.375±2.162		35.125±3.430	
Secondary education	78 (26.0)	10.167±0.702		33.179±0.727	
Higher education	207 (69.0)	14.198±0.457		33.652±0.457	
Educational background					
Related to health science/medical	23 (7.7)	14.043±1.200	0.671	34.130±1.857	0.889
Non-health science	57 (19.0)	12.719±0.730		34.070±1.043	
Not applicable	220 (73.3)	12.941±0.483		33.259±0.399	
Occupational status					
Working (full-time)	209 (69.7)	13.986±0.475	**<0.001**	33.564±0.467	0.417
Working (part-time)	15 (5.0)	8.400±1.704		31.466±0.919	
Housewife	50 (16.0)	10.820±0.815		33.320±0.964	
Retired	26 (8.7)	11.731±1.159		34.269±1.231	
Family income					
B40 (<RM 4360)	81 (27.0)	9.951±0.665	**<0.001**	32.876±0.787	0.101
M40 (RM 4360-9619)	154 (51.0)	13.9675±0.564		33.149±0.449	
T20 (>RM 9619 )	64 (21.7)	14.431±0.749		35.015±0.974	
Ethnicity					
Malay	284 (94.7)	12.849±0.404	**0.435**	33.581±0.395	**0.530**
Chinese	9 (3.0)	14.222±2.210		31.555±1.818	
Indian	7 (2.3)	16.857±1.738		31.857±1.078	
Ethnicity					
Urban	136 (45.3)	10.735±0.502		33.139±0.631	
Suburban	114 (38.0)	16.140±0.620	**<0.001**	33.728±0.514	**0.040**
Rural	50 (16.7)	11.900±0.962		33.840±0.942	

Educational level (p=0.029), occupation (p<0.001), family income (p<0.001) and residence (p<0.001) were significantly associated with the knowledge score. Conversely, marital status, educational background and ethnicity were not significantly associated with the knowledge score.

Age (p=0.007), marital status (p=0.011) and residence (p=0.040) were significantly associated with the attitude score. However, educational level, educational background, occupation, family income and ethnicity were not significantly associated with the attitude score.

### Correlation between knowledge and attitudes towards menopause

The correlations between the knowledge and attitude scores were assessed using Spearman’s rankorder correlation. A weak but statistically significant positive correlation was observed between knowledge and attitudes towards menopause (r = 0.249, p < 0.001), indicating that higher knowledge levels were associated with more positive attitudes.

## Discussion

While several studies have examined menopause-related knowledge and attitudes among Asian women, this study adds to the existing literature by providing community-based evidence from a local Malaysian setting among women aged 40–60 years. Unlike clinic-based studies, the present study reflects perspectives from women in the general community, making the findings particularly relevant to primary care and public health contexts. Furthermore, this study examined menopause-related knowledge and attitudes as well as their associations with sociodemographic factors, allowing for the identification of subgroups that may benefit from targeted educational and supportive interventions.

This study revealed that approximately half of the respondents had poor knowledge of menopause, with less than one-third demonstrating good knowledge. This aligns with the findings of other regional studies that reported limited awareness and understanding of menopause among Asian women.^7,10,23^ The knowledge section of the questionnaire used covered various aspects, including the definition of menopause, average age of onset, HRT, menopausal symptoms and associated risks. Deficits in knowledge were observed in multiple domains, particularly in the definition of menopause, typical age at menopause, role and benefits of HRT and recognition of menopause-related health risks such as osteoporosis, cardiovascular disease and diabetes. A similar trend was reported in the Asian Menopause Survey, which showed that only about half of participants were aware of HRT (54%), and fewer could specify its benefits (<30%).^34^

Limited knowledge ofmenopause may reduce women’s ability to recognise symptoms and associated health risks, which could influence health-seeking behaviours and preventive care.^9,10,19,35,36^ This knowledge gap may reflect limited emphasis on menopause-related education within routine primary care as well as reliance on informal or non-medical sources of information, which may provide inconsistent or inaccurate messages. Some symptoms related to menopause, such as vaginal dryness, heart discomfort, sleep-related problems, urinary issues and psychological challenges, may significantly affect women’s health. Recognising the health implications of menopause may help women to prepare mentally and physically for age-related changes. Furthermore, such understanding could support proactive health management by encouraging preventive measures, healthy habits and lifestyle modifications. Such knowledge may also increase awareness of early signs of health issues and help women to feel more confident in managing their health. In the present study, the limited awareness of osteoporosis and cardiovascular disease as menopause-associated risks suggests that some women may underestimate the importance of screening and early preventive care.^9,19,37,38^ Improving menopause-related health literacy may therefore enhance awareness and encourage healthier lifestyle practices and more proactive health-seeking behaviours.^9,22,39-41^

In this study, the respondents’ attitudes towards menopause appeared to be more negative than positive in several aspects. Some viewed menopauses as the end of a woman’s youth and the beginning of an unproductive life, perceiving it as an unpleasant experience accompanied by mental and emotional depletion. Such negative feelings may lead to anxiety and distress, potentially affecting psychosocial functioning and further reducing quality of life.^40-42^ These perceptions may be influenced by sociocultural norms that associate menopause with ageing, loss of femininity and reduced social value, particularly in Asian settings where menopause remains a sensitive or under discussed topic. For this reason, psychological interventions and educational programmes may help to support women during the menopausal transition and facilitate a smoother adjustment to this life stage.^9^

Despite the knowledge gap, some respondents expressed positive attitudes towards menopause. They reported that it made their lives easier, as they no longer needed to use sanitary pads, experience menstrual pain, manage irregular menstruation, use birth control methods or worry about pregnancy. These women viewed menopause as a normal stage in life that did not drastically alter their well-being and, in some cases, even made life more interesting. In comparison, a study of Sarawak women in Malaysia found that premenopausal women and those with higher educational levels held more positive views towards menopause. In that study, many participants expressed that while menopausal symptoms were unpleasant, the cessation of menstruation was a source of relief.^19^ Additionally, some Muslim women viewed menopause as an opportunity to fully engage in religious practices. These findings highlight the complex interplay between symptom experience, sociocultural beliefs and personal coping strategies.^5,9^

Several sociodemographic factors influenced the knowledge and attitude scores in our study. Higher educational level, occupational status and family income as well as urban residence were significantly associated with a higher knowledge score, while age, marital status and residence were significantly associated with the attitude score. These patterns are consistent with prior reports in Malaysia and internationally, which have linked educational attainment and socioeconomic status to greater health awareness and more positive perceptions of menopause.^10,19,43^ However, unlike several previous Malaysian studies that were conducted in clinical settings or focused on specific subgroups of women seeking healthcare, the present study involved a community-based sample drawn from the general population, including women who may not routinely engage with health services. This broader sampling context offers additional insights into menopause-related knowledge and attitudes at the community level.

This study found a significant, albeit weak, positive correlation between menopause-related knowledge and attitudes (r=0.249, P<0.01). This finding is consistent with previous reports suggesting that higher knowledge levels are associated with more positive attitudes towards menopause.^22,33,43^ Educational interventions targeting menopause have been shown in previous studies to improve knowledge and attitudes, which may contribute to better coping and reduced stigma.^22,41,43^

Our findings suggest that women with higher knowledge scores tend to report more positive attitudes towards menopause. This is consistent with a study involving middle-aged teachers, where educational interventions significantly improved attitudes towards menopause.^22^ Women who are well informed about menopause are generally better equipped to cope with its changes, and higher knowledge levels correlate with more positive attitudes, further supporting global evidence that education fosters adaptive coping.^20^

These findings suggest a potential need to strengthen public health initiatives addressing menopause awareness. Integrating menopause-related education into primary care, community outreach and digital media platforms can normalise discussion, address misconceptions and provide practical information on symptom management and healthy ageing.^41,44^ Healthcare professionals may also benefit from ongoing training to provide non-judgemental and culturally sensitive guidance to women transitioning through menopause.

### Limitations

The study has several limitations. Data collection was restricted to a single urban setting, which may not fully capture variations in menopausal experiences across different geographical regions in Malaysia, particularly in rural areas. Further, the use of convenience and snowball sampling via social media platforms may introduce selection bias and may limit the generalisability of the findings, particularly among women who are less digitally connected or from rural and minority communities. The predominance of Malay respondents does not fully represent Malaysia’s multi-ethnic population. In addition, the analysis was limited to bivariate statistical tests, and no multivariable analysis was performed; therefore, potential confounding factors could not be adjusted for when examining the associations between the sociodemographic characteristics, knowledge and attitudes. The cut-off scores used to categorise the knowledge and attitude levels were applied as practical analytical tools and do not represent universally validated standards. Reliance on self-reported data may also result in recall and social desirability bias, particularly regarding symptom severity and treatment-seeking behaviours. Consequently, the findings should be interpreted as community-specific and may not be nationally representative of all Malaysian women. Despite these limitations, the study provides important preliminary findings on menopause-related knowledge and attitudes among Malaysian women. Future studies should consider more representative sampling, and mixed method approaches to capture nuanced cultural and experiential aspects of menopause.

## Conclusion

Many women are still navigating the life changes associated with menopause. This study reveals that Malaysian women’s knowledge and attitudes towards menopause remain limited and may benefit from greater health education. Recommendations include expanding public health campaigns and educational programmes as well as increasing media coverage related to menopause. Integrating menopause-related education into primary care and community programmes may also be beneficial. Furthermore, there may be a need to address cultural narratives that equate menopause with lost femininity and to raise awareness of evidence-based symptom management strategies. Collectively, these efforts may contribute to improved public awareness of menopausal symptoms and available treatment options.

In conclusion, the observed correlation between knowledge and attitudes highlights a potential association that may inform targeted educational interventions. As this study was based on observational cross-sectional data, the findings should be interpreted as exploratory. Nevertheless, the findings suggest the importance of comprehensive menopause-related education, psychological support and a patient-centred approach to healthcare. These initiatives may support women in navigating the menopausal transition with greater knowledge, confidence and quality of life.

## Data sharing statement

Raw data are not available online. De-identified data are available upon reasonable request to the corresponding author.

**How does this paper make a difference in general practice?**

- Many midlife women in the community have limited knowledge about menopause and its associated health risks.
- The findings suggest that menopause-related topics may not be widely discussed or understood outside clinical settings, highlighting a potential role for primary care in improving awareness and providing more tailored counselling and reassurance during routine consultations.
- Community-based health education and primary care engagement may help to normalise conversations about menopause and support women in preparing for the menopausal transition.
